# Characteristics and Treatment Challenges of Patients With Comorbid ADHD and Psychosis: A Cross-Sectional Study in Early Intervention Services

**DOI:** 10.1192/bjo.2025.10499

**Published:** 2025-06-20

**Authors:** Salam Fahad, Nismen Lathif

**Affiliations:** Merseycare NHS Trust, Liverpool, United Kingdom

## Abstract

**Aims:** Comorbid attention deficit hyperactivity disorder (ADHD) and psychosis present significant challenges in Early Intervention in Psychosis (EIP) services. This study examined the prevalence, diagnostic patterns, and treatment challenges of this comorbidity within EIP teams in Halton and Warrington, UK.

**Methods:** A cross-sectional analysis of the current EIP caseload (N=180) was conducted, focusing on patients with suspected or diagnosed ADHD. Data on ADHD diagnosis, treatment status, antipsychotic medication use, and patient-reported outcomes were collected and analysed.

**Results:** Of the 180 EIP patients, 35 (19.4%) had suspected or diagnosed ADHD. Among these, 16 (45.7%) had a confirmed ADHD diagnosis, with only 8 (50%) receiving targeted ADHD treatment. No statistically significant differences were found in subjective quality of life or treatment satisfaction scores between patients receiving ADHD treatment and those not on treatment. The proportion of patients prescribed antipsychotic medication was similar between those on ADHD treatment (87.5%) and those not on ADHD treatment (88.9%).

**Conclusion:** This study reveals a high prevalence of comorbid ADHD in EIP services and significant gaps in diagnosis and treatment. The findings highlight the need for improved screening, integrated care pathways, and personalised treatment approaches for managing comorbid ADHD and psychosis. Future research should focus on developing evidence-based guidelines and exploring the impact of comprehensive intervention strategies on patient outcomes.

